# Imaging of Sacroiliac Pain: The Current State-of-the-Art

**DOI:** 10.3390/jpm14080873

**Published:** 2024-08-17

**Authors:** Marina Carotti, Luca Ceccarelli, Anna Claudia Poliseno, Francesca Ribichini, Francesca Bandinelli, Enrico Scarano, Sonia Farah, Marco Di Carlo, Andrea Giovagnoni, Fausto Salaffi

**Affiliations:** 1 Clinica di Radiologia, Dipartimento di Scienze Radiologiche, Azienda Ospedaliero-Universitaria delle Marche, 60126 Ancona, Italy; marina.carotti@gmail.com (M.C.); annaclaudia.poliseno@gmail.com (A.C.P.); francescaribichini@gmail.com (F.R.); a.giovagnoni@univpm.it (A.G.); 2 Radiology Unit, Department of Experimental, Diagnostic and Speciality Medicine, Sant’Orsola Hospital, University of Bologna, Via Albertoni 15, 40138 Bologna, Italy; luca.ceccarelli28@gmail.com; 3 Rheumatology Department, San Giovanni di Dio Hospital, USL Tuscany Center, 50143 Florence, Italy; francesca.bandi@gmail.com; 4 Department of Radiology, “San Carlo” Hospital, 85100 Potenza, Italy; enricoscarano@tiscali.it; 5 Rheumatology Unit, “Carlo Urbani” Hospital, Università Politecnica delle Marche, 60035 Jesi, Italy; sonia.farah91@gmail.com (S.F.); fausto.salaffi@gmail.com (F.S.)

**Keywords:** sacroiliac joint, sacroiliitis, magnetic resonance imaging, computed tomography

## Abstract

Pain in the sacroiliac (SI) region is a common clinical manifestation, often caused by diseases involving the SI joints. This is typically due to inflammation or degenerative changes, while infections or cancer are less frequent causes. The SI joint is challenging to image accurately because of its distinct anatomical characteristics. For an accurate diagnosis, conventional radiography often needs to be supplemented with more precise methods such as magnetic resonance imaging (MRI) or computed tomography (CT). Sacroiliitis, a common presenting feature of axial spondyloarthritis (axial SpA), manifests as bone marrow edema, erosions, sclerosis, and joint space narrowing. Septic sacroiliitis and repetitive stress injuries in sports can also cause changes resembling inflammatory sacroiliitis. Other conditions, such as osteitis condensans ilii (OCI), can mimic the radiologic characteristics of sacroiliitis. Inflammatory lesions are diagnosed by concurrent erosions, hyperostosis, and ankylosis. Ligament ossifications or mechanical stress can also result in arthritic disorders. Determining the exact diagnosis can be aided by the distribution of the lesions. Inflammatory lesions can affect any part of the articulation, including the inferior and posterior portions. Mechanical lesions, such as those seen in OCI, often occur in the anterior middle region of the joint. In cases of idiopathic skeletal hyperostosis, ligament ossification is found at the joint borders. This pictorial essay describes common SI joint problems, illustrated with multimodal imaging data. We, also, discuss strategies for selecting the best imaging modalities, along with imaging pitfalls, key points, and approaches for treating patients with suspected inflammatory back pain.

## 1. Introduction

Pain in the sacroiliac (SI) region is a common clinical manifestation frequently caused by diseases involving the SI joints. Approximately twenty-five percent of the cases of lower back discomfort are due to sacroiliitis, an inflammation of the SI joint, the largest axial joint in the human body. The SI joints are anatomically distinct, comprising a synovial joint component on the iliac side of the distal part of the joint, and cartilaginous and ligamentous components similar to those of a secondary cartilaginous joint [1].

The unique sigmoid shape and oblique orientation of the SI joints, along with the overlap of neighboring osseous structures, present challenges to planar imaging techniques such as conventional radiography or planar isotope bone scintigraphy. These challenges can make the interpretation of subtle changes difficult. Despite these difficulties, many individuals presenting with SI pain often undergo standard radiographs of the pelvis, lumbar spine, or SI joints.

According to the modified New York criteria, radiographic features such as erosions, sclerosis, and ankylosis are commonly observed in advanced inflammatory sacroiliitis and are graded from zero (normal) to four (ankylosis) [2]. However, many of these radiographic changes occur much later than the onset of symptoms, often not appearing for years after the initial presentation. Therefore, when imaging the SI joints, cross-sectional modalities such as magnetic resonance imaging (MRI), computed tomography (CT), and dual-energy CT (DECT) are more effective for early detection and accurate diagnosis. In some cases, it is necessary to supplement with nuclear medicine techniques such as positron emission tomography (PET) or single-photon emission computed tomography (SPECT).

Due to its multiplanar imaging capabilities and superior soft tissue and bone marrow contrast on short tau inversion recovery (STIR) and fat-saturated T2-weighted sequences—all crucial for identifying early inflammatory changes—MRI has become the preferred imaging modality for SI joint pathology [3]. The MRI examination of sacroiliac joints should be performed with patients in a supine position. An adequate visualization of both joint compartments demands sequences in two perpendicular planes, semi-coronal and semi-axial planes, which is a prerequisite for the precise location of joint abnormalities.

The current minimum standard for a diagnostic SIJ MR Includes at least four sequences: a fluid sensitive sequence, such as STIR or T2 FS (fat-saturated) sequence, in two perpendicular planes (semi-coronal and semi-axial), to detect bone marrow edema (BME); a marrow fat sensitive sequence, such as T1-weighted sequence, in the semi-coronal plane; a sequence designed for optimal visualization of the bone–cartilage interface (articular surface), such as a T1 FS (fat-saturated) sequence or a gradient echo sequence, depending on MR-equipment, in the semi-coronal plane. Optimally, the semi-axial field of view should be centered to enable additional visualization of the symphysis and the upper part of the hips, allowing the concomitant screening of changes in these areas, which could be affected in axSpA, and differential diagnoses.

More advanced MR sequences, such as DWI (diffusion-weighted imaging,) have been studied without confirming certain diagnostic advantages compared to the sequences previously mentioned.

The T2 Dixon technique has been used recently to diagnose acute sacroiliitis. In many instances, the Dixon approach is more accurate than the standard sequences using post-gadolinium sequences and offers a greater signal–noise ratio compared to STIR T2-weighted sequences. Using the fat- and water-only images, the Dixon method can also provide information about fatty deposition and sclerosis in chronic sacroiliitis [4,5]. For SI joint MRI, intravenous contrast is not necessary; however, it can enhance sensitivity when identifying soft tissue involvement or ongoing inflammation in probable infection cases.

Imaging SI joint pathology has benefited from technical advancements in musculoskeletal MRI, such as stronger, higher-resolution fluid-sensitive sequences, improved fat-suppression methods, and metal artifact-reduction sequences for postoperative imaging. The CT examination of sacroiliac joints should be performed with patients in a supine position, in a cranio-caudal way. An intravenous contrast agent is not required. Using DECT, the examination should be performed by two different energies (90 kV for tube A and 150 kV for tube B). From DECT acquisition, three different sets of images were obtained: low KVp images, high KVp images, and “mixed” images, which are obtained from the average of the two voltage data. 

The mixed series can be retro reconstructed with a high spatial algorithm (ex. at 0.6 mm) (bone filter), on which multi-parametric reconstruction (MPR) could be performed in the sagittal, coronal, and oblique planes. Post-processing image analysis by dedicated software, such as VNCa (virtual non-calcium), allows the detection of BME.

DECT methods have been reported more recently for detecting BME at acute fracture sites. These methods are comparable to fluid-sensitive MRI sequences in that they can identify areas of acute disease such as sacroiliitis [6]. The VNCa technique used by DECT to remove calcium from trabecular bone has been shown to be effective in detecting BME in patients with sacroiliitis who also have axial SpA [7,8].

The clinical picture and the radiologist’s or clinician’s access to imaging modalities will significantly impact the choice of imaging modality. In most cases, conventional radiography is the first-line modality and provides a good baseline for future comparison. However, the absence of radiographic alterations does not rule out the presence of an underlying inflammatory or infectious condition. As a result, many patients diagnosed with suspected inflammatory back pain may undergo further imaging, most often MRI, which provides an accurate assessment of anatomical changes and periarticular soft tissue structures [3,9].

For suspected infections, the preferred modalities are MRI with intravenous gadolinium contrast, planar SPECT, isotope bone scintigraphy, and PET. The stress fractures of the sacrum and pelvis can be detected by MRI, CT, and isotope bone scintigraphy, with the choice of modality depending primarily on personal preference and experience. When MRI is not appropriate, CT is useful as it can clearly distinguish between periarticular erosions, sclerosis, and osseous metastases.

Additionally, abdominal and pelvic CT imaging is frequently performed for other clinical scenarios, often leading to the incidental discovery of SI joint pathology.

This several common SI joint diseases with multimodal imaging data as examples. We also discuss approaches to managing patients with suspected inflammatory back pain, along with tips and tricks for selecting the best imaging modalities.

## 2. Inflammatory Sacroiliitis

A key element of axial SpA, which encompasses undifferentiated SpA, reactive arthritis, psoriatic arthritis, inflammatory bowel disease-associated SpA, and ankylosing spondylitis (AS), is inflammatory sacroiliitis. Although there is considerable overlap among these disorders, SI joint involvement is a common feature. Inflammatory back pain (IBP) is chronic pain concentrated in the axial spine and sacroiliac joints, distinguishing it from mechanical back pain through specific diagnostic features. Recall that the characteristic features of IBP include age of onset less than 40, back pain duration exceeding 3 months, insidious onset, morning stiffness, and improvement with exercise [10]. In contrast, the causes of mechanical back pain typically exacerbate with movement and exercise and often correlate with injury, presenting a more acute onset. Clinicians must recognize these distinctive features for early diagnosis and appropriate management. A referral to a rheumatology specialist may be warranted after establishing a diagnosis of IBP using accepted criteria (Table 1), particularly when accompanied by positive HLA-B27 testing and the confirmation of sacroiliitis through imaging.

The pattern of the involvement of SI joints can be unilateral or bilateral and may range from mild to severe inflammation, leading to partial or complete ankylosis. In 85% to 90% of the patients with AS, sacroiliitis is typically bilateral and symmetrical [11]. However, most SpA subgroups eventually progress to AS. Other subgroups are less frequently bilateral and tend to affect the SI joints unilaterally or asymmetrically.

Extra-articular manifestations, such as psoriasis, uveitis, and inflammatory bowel disease, are also common in SpA. Additionally, the condition includes extra-axial presentations such as enthesitis and/or peripheral articular involvement [12,13]. Due to these varied presentations, diagnosis can sometimes be challenging. Imaging results, along with other clinical symptoms, play a crucial role in diagnosis. Axial SpA is traditionally diagnosed using a combination of clinical symptoms and definitive radiographic damage, either radiographic sacroiliitis (based on the modified New York criteria) or syndesmophytes in the spine [14,15].

However, this type of structural damage does not show up for years after the first manifestation of the disease, which causes a major delay in diagnosis [15,16]. In addition, the reliability of sacroiliitis on radiographs has been consistently reported as poor, regardless of the reader (rheumatologist or radiologist) or the type of reading (local reading or central reading) [17,18]. In patients with axial SpA, MRI became available in the late 1990s to assess the presence of inflammation in the spine and SI joints. Even in patients without structural damage, inflammation can be found, indicating that inflammation may be the initial stage of a sequence that eventually leads to radiographic progression. Since then, MRI has been used to diagnose patients with axial SpA. The Assessment of SpondyloArthritis international Society (ASAS) group has presented several definitions of what constitutes a “positive” MRI of the SI joint [19,20] (Table 2). The ASAS classification criteria for axial SpA [21], which have been validated in many populations [20,21,22], include these abnormalities (radiographic sacroiliitis and sacroiliitis on MRI) as entry criteria (Table 3). 

**Figure 1:**
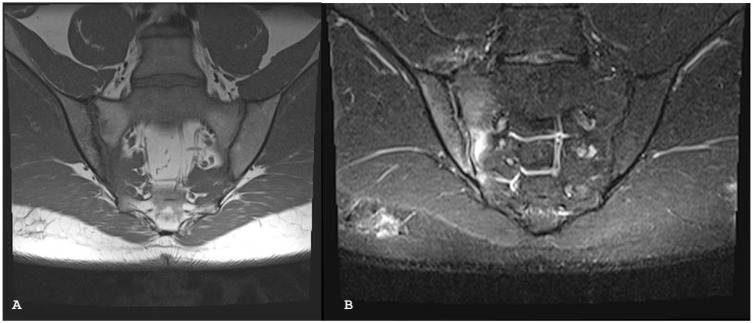
Oblique coronal T1-weighted magnetic resonance imaging (**A**) and oblique coronal short tau inversion recovery (**B**) sequences show periarticular bone marrow edema involving the right sacroiliac joint associated with multiple subchondral erosions, the confluence of which has resulted in the pseudo-widened appearance of the right sacroiliac joint. Capsulitis can be visible anteriorly (**B**). Note that the erosions are more on the iliac, rather than the sacral, side of the joint because the articular cartilage overlying the iliac side is half as thin as that on the sacral side.

These lesions have been consistently associated with axial SpA. BME, synovitis, capsulitis, and enthesitis are the MRI features of inflammation (Figure 1). 

**Figure 2:**
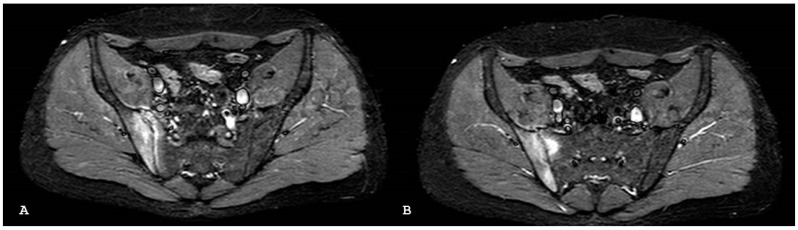
Oblique axial short tau inversion recovery images (**A**,**B)** show subchondral bone edema on both sides of the right sacroiliac joint in patient with psoriatic arthritis.

The only prerequisite for the diagnosis of active sacroiliitis is BME [20], but this criterion must be present substantially enough to allow the diagnosis of sacroiliitis. For BME to be considered substantial, it must have a depth of ≥1 cm and be discernible on a minimum of two consecutive MRI images, or at two different sites on the same image [23,24] (Figure 2). 

**Figure 3:**
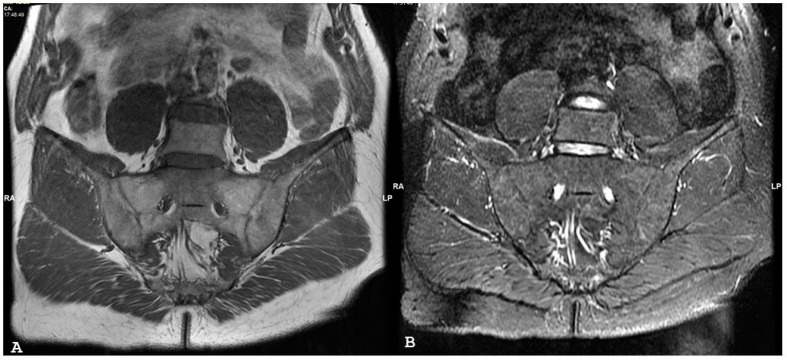
Oblique coronal T1-weighted magnetic resonance imaging (**A**) demonstrates subchondral fatty replacement and bony fusion consistent with end-stage ankylosis from previous bilateral sacroiliitis. Corresponding oblique coronal short tau inversion recovery sequence (**B**) shows lack of bone marrow edema.

**Figure 4:**
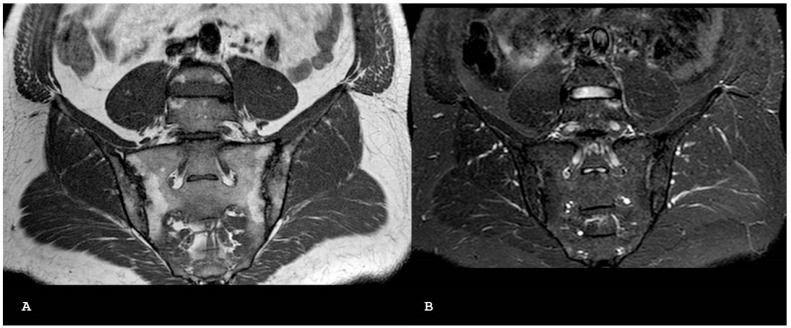
Oblique coronal T1-weighted imaging (**A**) demonstrates bony irregularity with multiple subchondral erosions on both the sacral and iliac sides and pseudo-widened appearance of both sacroiliac joints. New fat metaplasia in the subchondral bone marrow of the sacral bone is also evident. These findings are suggestive of chronic sacroiliitis. Oblique coronal short tau inversion recovery (**B**) demonstrates the non-active involvement of both sacral–iliac joints.

Synovitis, enthesitis, joint space fluid, and capsulitis are compatible with SpA-related sacroiliitis but are not considered specific enough to support a diagnosis of sacroiliitis because they are uncommon as a single MRI finding without the concomitant presence of BME [20]. Subchondral sclerosis, erosions, joint space narrowing, and the ankylosis of the SI joint are examples of structural changes indicative of long-lasting disease (Figure 3 and Figure 4).

**Figure 5:**
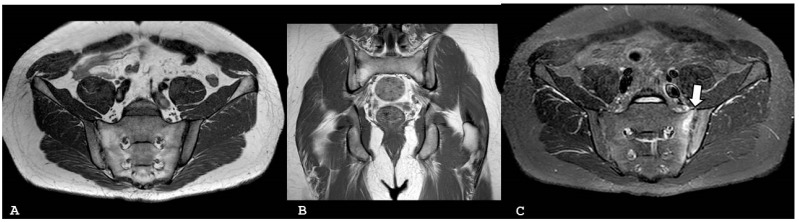
Oblique coronal (**A**) and coronal (**B**) T1-weighted magnetic resonance imaging sequences show that the excavated iliac bone of the right sacroiliac joint is replaced by tissue showing MR signal intensity similar or higher than normal bone marrow (backfill). New fat metaplasia in the subchondral bone marrow of the right sacral and iliac bones is also evident. Oblique coronal short tau inversion recovery (**C**) sequence demonstrates abnormal signal intensity in marrow adjacent to the left sacroiliac joint suggestive of sacroiliitis. Capsulitis can be visible anteriorly (arrow). Note synovitis with increased signal intensity in the joint space of the left sacroiliac joint. The non-active involvement of the right sacroiliac joint.

**Figure 6:**
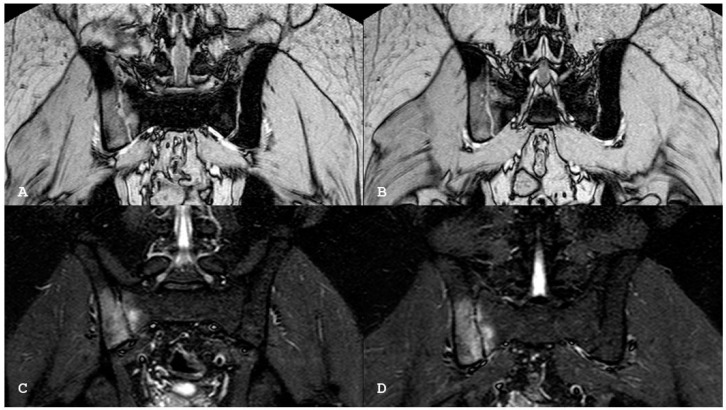
Oblique coronal gradient echo T2 sequences (**A**,**B**) and oblique coronal short tau inversion recovery (**C**,**D**) magnetic resonance imaging sequences show bony irregularity with subchondral erosions associated with the subchondral bone edema of both the sacral and iliac sides of the right sacroiliac joint.

The “backfill” is an intermediate stage between erosion and ankylosis, defined as reparative fatty metaplasia next to the SI joints, which are surrounded by sclerosis. It is possible that this biomarker is more specific for SpA than previously thought [25] (Figure 5). However, in addition to BME, erosions and structural lesions should also be considered, as they increase the specificity of the diagnosis of inflammatory sacroiliitis [26,27] (Figure 6).

It is easier to identify individuals with axial SpA early with MRI rather than radiography since inflammatory signs are detectable before structural damage [21,28]. From 20 to 42% of all the patients with axial SpA have active sacroiliitis on MRI [29,30,31,32].

However, even subjects without axial SpA may present with MRI signs suggestive of sacroiliitis. According to a study by Weber et al., of 59 healthy participants, up to 22% revealed evidence of sacroiliitis on MRI [33]. Arnbak et al. showed that according to the ASAS criteria, 21% of 1020 unselected individuals with persistent back pain had sacroiliitis on MRI [34]. It might be advisable for clinicians not to over-rely on a positive MRI for the purpose of axial SpA diagnosis since the specificity of SI joint MRI for inflammatory sacroiliitis is unknown [35].

De Winte et al. compared SI joint MRIs in patients with axial SpA who had a positive MRI of the SI joints after central reading, in patients with chronic low back pain, in habitual runners, and in women with postpartum low back pain in order to understand the prevalence and extent of SI joint inflammation in healthy individuals and in those with known mechanical stresses acting on the SI joints [36]. The researchers concluded that a substantial proportion of the healthy, asymptomatic individuals, runners, and women with postpartum back pain may have positive MRI findings of the SI joint that are highly suggestive, but not reflective, of axial SpA. Patients with axial SpA have more extensive lesions, as reflected by Spondyloarthritis Research Consortium of Canada (SPARCC) MRI index scores ≥ 5 and the presence of deep lesions), than healthy, asymptomatic individuals [37].

More recently, cut-offs have been proposed for active and structural lesions defined as typical of axial SpA. Specifically, BME on MRI is considered highly suggestive of axial SpA if present in at least four quadrants of the SI joint in any location or in the same locations on at least three consecutive slices of the SI joint. A positive MRI for structural lesions in the SI joint includes at least three quadrants of the SI joint with erosion or in the same location in two consecutive slices or the presence of fat lesions in at least five quadrants of the SI joint or in three consecutive slices. MRI cut-offs for active and structurally defined lesions typical of axial SpA have very high positive predictive value for the clinical diagnosis of axial SpA. The ASAS-MRI Working Group consensus outlined several general recommendations regarding the optimal interpretation of MR injuries in the sacroiliac joint (Table 1) [19]. 

Compared to adults, diagnosing sacroiliitis on MRI in pediatric populations is challenging mainly due to normal variability in the maturing pediatric sacroiliac joint. The normal pediatric sacroiliac joints commonly display a subchondral rim of increased signal on fluid-sensitive sequences also referred to as subchondral flaring. Subchondral “flaring” usually occurs symmetrically, is more prominent on the sacral side, and vanishes with skeletal maturity, usually around the ages of 15 for girls and 17 for boys.

If not aware of these normal signal changes, a false-positive diagnosis of sacroiliitis could be made, since they can mimic BME. When there is a distinct difference between the left and right sides, when the high T2/STIR signal is only seen at the iliac side, or if there is a definite high T2/STIR signal of any pattern in teenagers with closed sacral apophyses, suspicion for true BME and sacroiliitis should be raised. Compared to adults, children’s BME is more specific for sacroiliitis. Moreover, in children, a detectable amount of fluid that can exist in a normal sacroiliac joint is not and should not be interpreted as a pathological finding. Another possible pitfall to avoid when reading sacroiliac joint MRI is the misinterpretation of vascular channels abutting the superior edge of the joint, such as capsulitis. Structural lesions of sacroiliitis are less frequently seen in children. Furthermore, erosions are very difficult to depict on T1 images due to the normal variation. If seen, they are highly specific for juvenile SpA [38].

## 3. Infective (Septic) Sacroiliitis

Pyogenic sacroiliitis is a relatively uncommon condition, accounting for only 1–2 percent of the cases of septic arthritis according to recent studies [9,39]. This condition is associated with low clinical suspicion, an ambiguous clinical presentation, and poorly localized symptoms, making diagnosis challenging [40]. Staphylococcus aureus and Pseudomonas aeruginosa are the primary causative pathogens due to their frequent association with bacteremia [41,42].

Infectious sacroiliitis should be considered in patients presenting with unilateral sacroiliitis and associated symptoms such as fever, leukocytosis, elevated inflammatory markers, or bacteremia, especially when there is soft tissue involvement. Pathogens typically reach the SI joints hematogenously, although local extension from nearby soft tissue or bone is also possible. Risk factors for infective sacroiliitis include infectious endocarditis, seeding from other infection sites, joint injections, trauma, and other conditions [43,44].

**Figure 7:**
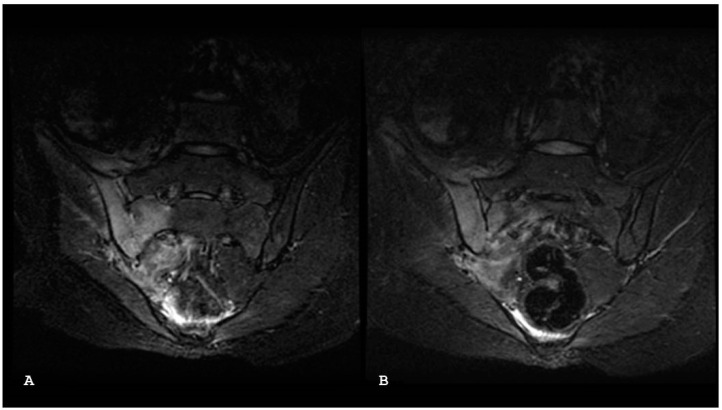
Oblique coronal short tau inversion recovery magnetic resonance imaging sequences (**A**,**B**) show diffuse bone marrow edema on both sides of the right sacroiliac joint, associated with capsulitis and enthesitis and with inflammatory fluid in the para-rectal adipose space. The spread of inflammation involving the peri-articular soft tissues supports the diagnosis of infective sacroiliitis.

**Figure 8:**
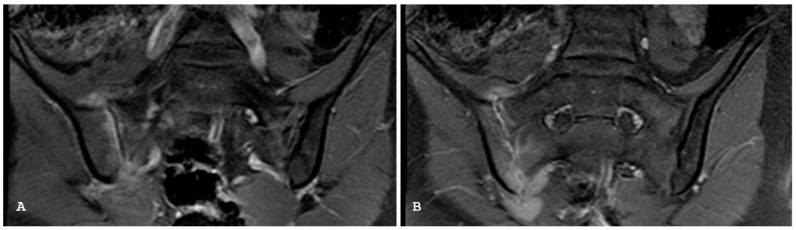
Oblique coronal T1 fat-saturated after gadolinium contrast magnetic resonance imaging sequences (**A**,**B**) demonstrate diffuse osteitis with associated synovitis, capsulitis, and periarticular inflammatory abscess in a 12-yr-old girl with clinic-laboratory signs of inflammation.

Radiographic changes may take days or weeks to appear following the onset of symptoms, so a negative initial radiograph should not reassure the physician. When infection is suspected, additional imaging with MRI and CT is recommended. Prior to the widespread use of MRI, isotope bone scintigraphy was commonly employed. However, MRI is now considered the best imaging modality as it clearly reveals erosive changes, BME within the SI joint and surrounding bones, effusion, and the extent of soft tissue involvement and potential spread to muscles such as the obturator internus, piriformis, and iliacus muscles (Figure 7 and Figure 8).

In axial SpA, inflammation is confined to the bone and sacroiliac joint space, without extending to the extracapsular soft tissue. Thick capsulitis is more commonly observed in infectious sacroiliitis than in axial SpA. Both infectious sacroiliitis and sacroiliitis associated with SpA can present with subchondral or periarticular erosions, complicating the differential diagnosis.

All the suspected cases of infectious sacroiliitis should routinely undergo intravenous gadolinium contrast-enhanced imaging, which is particularly useful for detecting abnormal soft tissue enhancement and abscess formation [43]. When pelvic discomfort and fever are present, CT scans can be valuable in an emergency to rule out gynecologic or gastrointestinal infections. The early CT indicators of septic sacroiliitis include muscle edema, anterior sacroiliac capsule bulging, and fat infiltration anterior to the sacroiliac joint [45].

MRI is the preferred imaging method for diagnosing this condition, although CT may be necessary in certain situations. Additionally, PET using fluorine-18 fluorodeoxyglucose appears to be a promising technique for the very early detection of infective sacroiliitis, even before abnormalities are visible on MRI or CT scans [46].

## 4. Postpartum Bone Marrow Edema at the Sacroiliac Joints

Women frequently experience low back discomfort both during and after pregnancy [47]. Typically, this pain develops during pregnancy and resolves within the first six weeks postpartum [48]. However, approximately 4% of women continue to suffer from back pain for more than six months after giving birth [49]. There is a wide range of differential diagnoses for low back pain during pregnancy and the postpartum period. The pubic symphysis and the sacroiliac joints are the primary musculoskeletal structures in the pelvis affected during these times [50].

The term “pelvic girdle syndrome” describes daily pain in both the sacroiliac joints and the pubic symphysis. According to Albert et al., 21% of pregnant women with pelvic girdle syndrome continued to experience symptoms two years after giving birth [51]. If only the sacroiliac joints were affected, up to 5% of patients experienced symptoms up to two years postpartum. Women with persistent pain typically undergo sacroiliac joint MRIs. In a follow-up investigation, 3.8% of the women with MRI-diagnosed low back discomfort related to pregnancy also had SpA [52]. This proportion is somewhat higher than the reported prevalence of SpA in the United States (1–1.4%) and Europe (0.3–2.5%), and it is close to the upper range of inflammatory back pain prevalence (up to 3.4%) [53,54,55].

Hormonal changes combined with increased mechanical stress during pregnancy are the most typical causes of this discomfort [55]. For instance, the hormone relaxin, released by the reproductive organs, affects various bodily systems, including the musculoskeletal system. Relaxin levels rise during pregnancy, loosening pelvic ligaments in anticipation of childbirth [56,57,58].

Lower back discomfort during the postpartum phase may also be caused by an infection in the SI joints, even if is uncommon [46]. Stress fractures in the sacrum have also been documented following labor [59]. Additionally, inflammatory conditions like SpA might manifest during pregnancy or the postpartum period.

**Figure 9:**
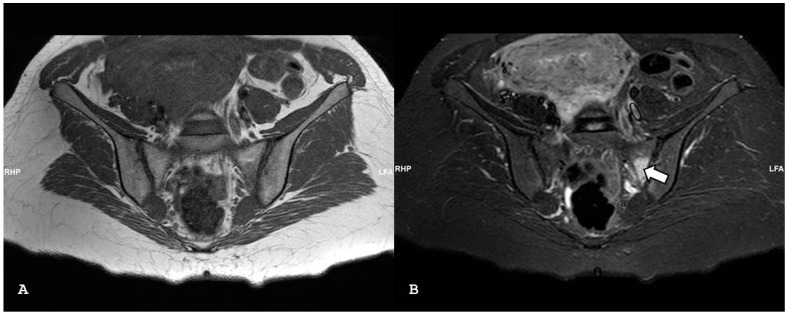
Oblique coronal T1-weighted (**A**) and oblique coronal short tau inversion recovery (**B**) magnetic resonance imaging sequences show focal patchy edema (arrow) at the mid-sacral area close to the left sacroiliac joint, in a woman who has recently given birth. No fracture line is evident.

An MRI of the SI joints can reveal bone marrow abnormalities associated with these conditions. However, there is uncertainty about distinguishing between mechanically induced postpartum bone marrow abnormalities and MRI-diagnosed inflammatory sacroiliitis. Imaging findings typical of SpA at the sacroiliac joints include subchondral erosions and sclerosis, fatty bone marrow replacement at the sites of previous sacroiliitis, BME surrounding the SI joint as a sign of active inflammation, and ultimately, the ankylosis of the SI joints [4]. BME in women who have recently given birth may resemble acute sacroiliitis (Figure 9). However, pregnancy does not appear to have an impact on the persistence of BME in the long term [60].

Anatomical observations such as fatty bone marrow replacement or erosions can help distinguish between inflammatory sacroiliitis and bone marrow changes caused by hormonal influences and mechanical stress. Agten et al. discovered that 63.3% of women had pregnancy-induced BME at the SI joints in the early postpartum phase [61]. This condition may be mistaken for sacroiliitis associated with axial SpA, as it results from prolonged mechanical stress. When BME is identified in women who have recently given birth, the degree and location of MRI changes make it challenging to differentiate this condition from inflammatory sacroiliitis. This observation suggests that BME presenting as inflammatory changes may be due to persistent mechanical stress on the sacroiliac joints. However, structural alterations such as fatty bone marrow replacement and erosions are more common in axial SpA and rarely observed in the postpartum, as expected given the underlying pathology. Interestingly, in the postpartum patients, there was no correlation between BME and pain. The SPARCC MRI index score showed no correlation between the extent of BME and pain intensity. 

Other studies have documented MRI findings in postpartum women. Wurdinger et al. scanned 19 postpartum individuals and found only 1 patient with a sacroiliac joint lesion, diagnosed as a stress fracture [62]. They focused on changes in the pubic symphysis and did not use STIR sequences. In contrast, Hermann et al. used an STIR sequence and observed signal intensity changes around the sacroiliac joints in 62% of asymptomatic postpartum women, similar to the findings in our postpartum group. Their study included 21 asymptomatic postpartum women and 35 patients with postpartum pelvic pain. Among symptomatic postpartum women, 66% had BME at the sacroiliac joints [63].

Another study involving the MRI scans of 93 pregnant or postpartum women found no structural abnormalities such as erosions or fatty bone marrow replacement [52]. In conclusion, when interpreting an MRI of the SI joints, it is crucial to consider the obstetric history of women suspected of having axial SpA [64].

## 5. Osteoarthritis

Although underdiagnosed and underestimated, SI joint osteoarthritis is common in elderly adults [65]. This condition typically affects the iliac side of the joint more, as it bears more weight and has less articular cartilage. Osteoarthritis and related changes are particularly frequent in patients with scoliosis or developmental anomalies, such as transitional vertebrae or accessory joint facets, and are typically located in the anterior part of the mid-third of the SI joint, which is the area where mechanical stress is concentrated. 

Imaging findings best confirmed by CT include joint-space narrowing to less than 2 mm, subchondral sclerosis (which is usually dense, well defined, and variable in thickness), and osteophyte formation. Additionally, intra-articular air (vacuum phenomenon), subchondral cysts, and ankylosis may occasionally be observed.

It is important to distinguish between the para-articular bony ankylosis caused by bridging osteophytosis in osteoarthritis and the actual intra-articular bony ankylosis seen in axial SpA [66]. Although not as commonly seen as erosions in axial SpA, small erosion-like lesions have occasionally been reported in osteoarthritis.

**Figure 10:**
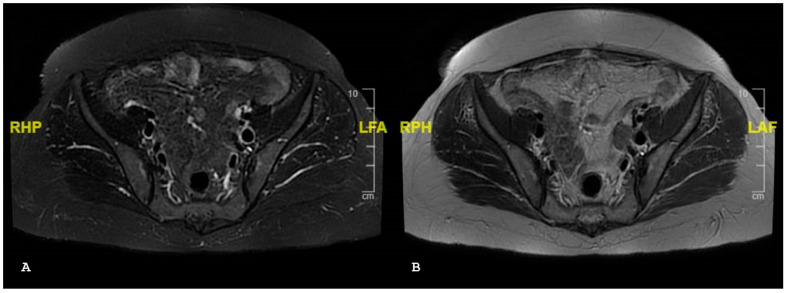
Axial short tau inversion recovery (**A**) and axial T1-weighted magnetic (**B**) resonance imaging sequences show moderate subchondral sclerosis (a map morphology), surrounded by a thin rhyme of bone edema.

In addition to the changes best documented by CT, MRI may also detect dispersed fat deposition and subchondral BME, which usually occurs at the anterior portion of the SI joint adjacent to sclerotic areas, directly in the subchondral area, or in areas with osteophytes (Figure 10).

**Figure 11:**
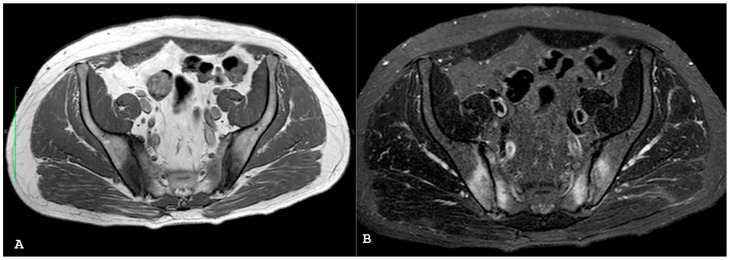
Axial T1-weighted (**A**) and axial short tau inversion recovery (**B**) magnetic resonance imaging sequences show bilateral periarticular bone marrow edema consistent with active sacroiliitis associated without bony irregularity.

BME is usually minimal, noticeable on only one or two slices, and typically less pronounced than in inflammatory sacroiliitis [65] (Figure 11). Osteoarthritis is frequently accompanied by subchondral fatty alterations that resemble Modic type II endplate changes and can mimic inflammatory “backfill.”

## 6. Osteitis Condensans Ilii

One rare cause of persistent back pain is osteitis condensans ilii (OCI), which continues to be a medical mystery [67]. With an incidence of 0.9–2.5%, OCI is a disorder of the SI bones in the pelvic cavity [68]. It is often discovered incidentally during pelvic imaging in individuals with low back pain or those who are otherwise asymptomatic [69]. OCI is frequently reported in pre- or postpartum women of childbearing age [70]. The clinical course of OCI includes both asymptomatic and symptomatic variants, and when symptomatic, it accounts for roughly 1–2.5% of the lower back pain cases [68]. Similar to axial SpA, it can present as lower back pain at an earlier age in some individuals [71]. Sclerosis is usually restricted to the iliac bone adjacent to the SI joints. OCI is a benign condition associated with lax ligaments and mechanical stress across the SI joints [69].

Due to limited awareness among primary care physicians regarding the clinical features, progression, diagnostic modalities, and treatment principles of this disease, there is a risk of misdiagnosis and the overuse of multiple, sometimes unnecessary diagnostic tests, driving up healthcare costs. OCI is hypothesized to result from mechanical tension and imbalance across the SI joints, although its precise cause remains unknown [68]. Diagnosis is based on the presence of characteristic sclerotic lesions on SI radiographs after ruling out other disorders associated with back pain [67]. Management typically involves conservative measures such as physical therapy and painkillers. The prognosis is generally favorable. OCI is usually discovered incidentally on routine X-rays when no musculoskeletal symptoms are present.

First reported in 1926 by Sicard et al. [72], the disorder was later named “osteitis condensans ilii” by Barsony and Polgar [73] after they described 15 patients with lower back pain, SI joint tenderness on physical examination, and bilateral or unilateral sclerotic iliac lesions on plain radiographs. Despite numerous studies since its initial description, the pathophysiology of OCI remains unclear [68]. OCI is most prevalent in the prepartum or postpartum phase in young women of childbearing age, but can also manifest in men and nulliparous women. In these cases, it is frequently associated with obesity or excessive physical strain on the SI joints, such as that caused by scoliosis [71,74].

**Figure 12:**
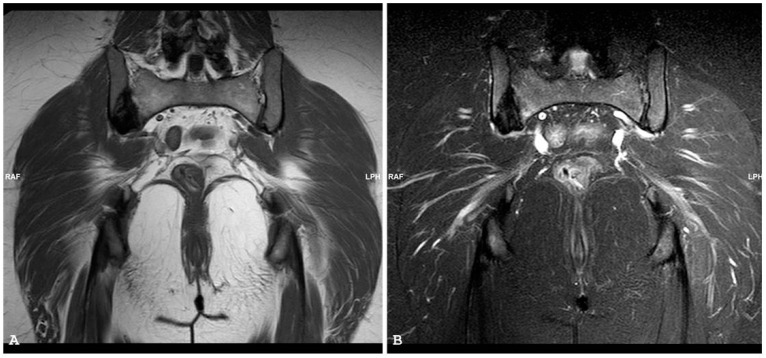
Coronal T1-weighted (**A**) and coronal short tau inversion recovery (**B**) magnetic resonance imaging sequences show the triangular areas of sclerosis along the inferior iliac margins of the right sacroiliac joint.

OCI is frequently misdiagnosed as sacroiliitis [69]. Low back pain may be an early symptom of OCI. Radiological findings (X-rays and CT) provide the ability to differentiate OCI from axial SpA [75]. The typical radiographic findings of OCI include a triangular-shaped area of sclerosis with the apex pointing upward along the iliac bone’s subcortical articular surface and extending into the surrounding medullary cavity (Figure 12). These results are generally symmetrical.

OCI can be distinguished radiologically by observing joint space narrowing in the SI joints on plain radiographs, along with the absence of erosions and ankylosis [69]. The use of MRI technology to evaluate OCI was first proposed in 1994 [76]. On MRI, the regions of subchondral sclerosis in the iliac portion of the joint are usually located in the anterior part, primarily on the iliac side. These regions appear as low signal intensity on T1 and T2 weighted imaging (Figure 12). Although some OCI cases may show BME on SI joint MRI, BME is not typically linked to this condition [71].

Differentiating OCI from early axial SpA may be facilitated by closely examining the position and distribution pattern of BME. In axial SpA, BME is typically discontinuous and extends to areas of the joint outside the load-bearing regions, whereas in OCI, iliac BME generally has a distinct continuous distribution and is positioned peripherally to the subchondral sclerosis [71]. BME lesions in OCI are typically found in the anterior region of the SI joints, while axial SpA lesions are more commonly located in the central region of the joint [71]. OCI does not exhibit any periosteal response or localized soft tissue abnormalities on MRI [76,77].

CT of the SI joints is considered the gold standard for detecting structural deterioration, including erosions, sclerosis, and ankylosis, due to its sensitivity. Attenuation coefficients, which measure the degree to which X-rays can penetrate a material [78], are used to assess the subchondral bone at the site of sclerosis in conditions such as OCI, diffuse idiopathic skeletal hyperostosis (DISH), AS, and osteoarthritis. The average bone mineral density, expressed in Hounsfield units (HUs), of a region of interest (ROI) in the trabecular bone (excluding cortical bone) corresponds to the subchondral bone attenuation coefficient. The estimation of the attenuation coefficient, performed using DECT, may reasonably allow the replacement of MRI with the aforementioned method for the diagnosis of OCI [78,79,80,81,82,83].

## 7. Diffuse Idiopathic Skeletal Hyperostosis

Anterior vertebral body and peripheral entheses can exhibit pathological calcification and ossification in diffuse idiopathic skeletal hyperostosis (DISH), a non-inflammatory disease characterized by the bony bridging of several vertebral bodies [84]. The prevalence of DISH varies, ranging from 2.9% in the Asian population over 50 years old to 42.0% in European men over 65 years old [85,86]. Reported risk factors for DISH include advanced age, male gender, and metabolic conditions such as obesity, hypertension, and type 2 diabetes mellitus [87].

DISH is a pathological condition marked by the progressive ossification of ligaments at their attachment sites and throughout the body, leading to spinal ankylosis due to the ossification of the anterior longitudinal ligament. Initially described by Forestier et al. in 1950 and later named “DISH” by Resnick et al., the disease is also known as Forestier’s disease with extraspinal manifestations [88]. Resnick and Niwayama et al. established the most widely used diagnostic criteria for DISH [89]. According to these criteria, a diagnosis of DISH can be made if the following conditions are met: [1] ankylosis of the SI joint; [2] bony bridging of the anterior or lateral aspect of at least four vertebral bodies; and [3] largely preserved intervertebral disk height.

**Figure 13:**
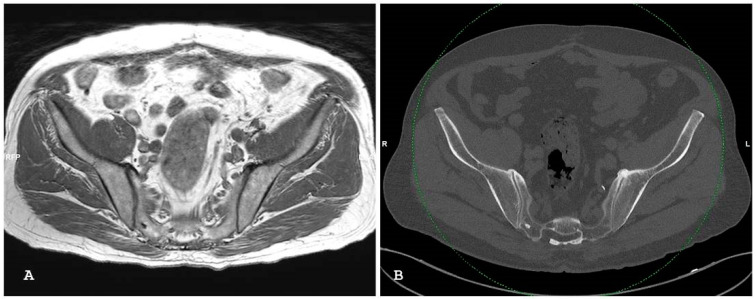
Oblique coronal T1-weighted magnetic resonance imaging sequence (**A**) shows entheseal bridging anterior to the sacroiliac joints. Axial computed tomography with bone window (**B**) shows bony bridging anterior to the sacroiliac joints. There are no subchondral erosions or sclerosis.

DISH is not confined to the spine; extraspinal symptoms include hyperostosis at the rotator cuff, hand, the deltoid tuberosity of the humerus, pelvis, ulnar olecranon, and patella [90,91]. Research by Leibushor et al. indicates that SI joint fusion, anterior and posterior bridging, and entheseal bridging are significantly more common in individuals with DISH compared to those without the condition [92] (Figure 13).

These characteristics can also be utilized to identify DISH patients and differentiate them from non-DISH individuals. In certain cases, the diagnosis of DISH may be complicated by the presence of SI joint fusion or ankylosis, suggestive of the coexisting presence of AS [93]. Patients with both DISH and AS may share a similar pathogenetic pathway, leading to inflammatory-related enthesitis in younger individuals, potentially linked to the human leukocyte antigen (HLA)-B27 positivity, and a more mechanically related enthesopathy in older patients if SI joints fusion is present. The idea that SI joint intra-articular involvement rules out the diagnosis of DISH is challenged by the occurrence of joint bridging and fusion in DISH, similar to that seen in patients with AS, contrasting sharply with this exclusion criterion of the Resnick DISH classification criteria [88,89]. In individuals with DISH, SI joint fusion is not typically seen; instead, only minor synovial area narrowing, and ligamentous region obliteration may occur [94]. 

While the Resnick criteria do not require SI joint involvement for a DISH diagnosis, other investigations have found that 23% of the DISH cases are linked to fusion [92,95]. According to a study in 2017, SI joint fusion in patients with both DISH and AS may be caused by similar developmental pathways, resulting in more mechanistically associated enthesopathy in older DISH patients and inflammation-induced enthesitis in younger AS patients [92].

Partial and total SI joint fusion was found to be more common in AS than in DISH according to Takahashi et al.; in DISH, patients exhibited partial or complete SI joint fusion in as many as 63% of the cases [96]. Some studies have reported that 16% to 30% of DISH cases are linked to mild BME and fatty marrow metaplasia; however, erosions and subchondral sclerosis are uncommon, distinguishing these changes from axial SpA [94,97].

## 8. Insufficiency (Stress) Fractures

Low back pain could be caused by injury to the SI joints. Approximately 88% of SI joint injuries are caused by either acute trauma or recurrent microtrauma. Sports injuries involving the SI joints are very common, and prolonged stress in athletes may result also in reactive alterations to the soft tissues. Four percent of the cases are idiopathic and 20% are connected to pregnancy [98].

**Figure 14:**
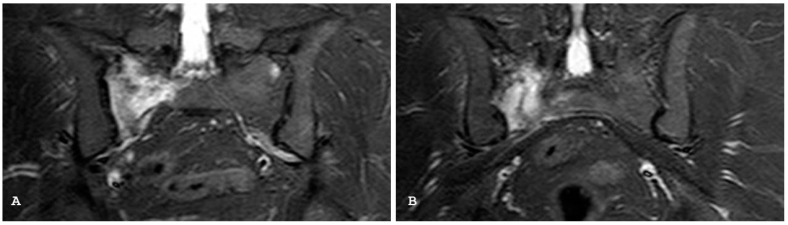
Oblique coronal short tau inversion recovery magnetic resonance imaging sequences (**A**,**B**) show diffuse bony edema of the right sacral side, with thin hypointense stria with an oblique vertical course. The sacroiliac joint is regular without edema of the iliac side.

One type of SI joint damage is trauma related to pelvic ring injuries. There are three primary types of pelvic ring traumatic injuries: vertical shear-type injuries, lateral compression injuries, and anteroposterior compression injuries (Figure 14). Additionally, there are three types of SI joint injuries: complete, partial, and SI fracture–dislocations. These injuries are associated with three different kinds of fractures connected to SI joint damage: (1) type 1 fracture: an anterior aspect of the S2 foramen minor fracture, producing a sizable, stable crescent-shaped piece. This type involves the least amount of ligament damage and affects less than one-third of the SI joint; (2) type 2 fracture: occurs independently between the anterior side of the S1 and S2 foramen; (3) sacral insufficiency fractures (SIFs) are a form of stress fracture that occurs when normal load is applied to bone that has lost some of its flexibility. SIFs are more frequent in elderly women and are typically associated with underlying metabolic bone diseases such as osteoporosis or Paget’s disease, or in the setting of prior pelvic irradiation [99,100]. These fractures are typically bilateral and vertical, passing through the sacral alae and paralleling the SI joints, often coupled with a transverse component resembling the capital letter H, hence the terms “Honda sign,” “H sign,” or “H pattern.”

**Figure 15:**
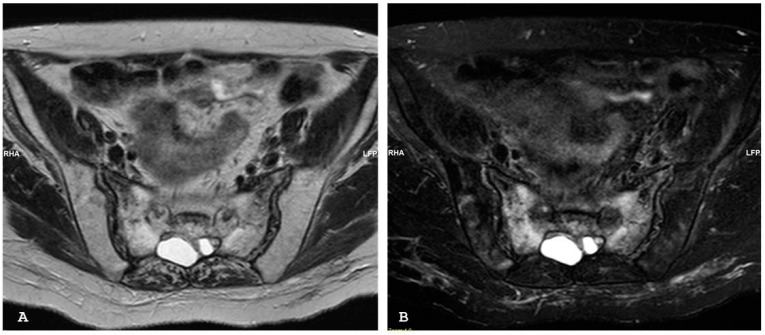
Axial T2-weighted (**A**) and axial short tau inversion recovery (**B**) magnetic resonance imaging sequences show hypointense fracture lines with surrounding acute bone marrow edema within the sacrum. Note the sacral Tarlov perineurial cysts.

The iliac bones close to the SI joints might also cause pain, although they are less frequently involved [100]. Fracture lines are difficult to see on traditional radiographs; therefore, MRIs, CT scans, or isotope bone scintigraphy are usually used to diagnose fractures (Figure 15). On imaging, vertical lucent lines on both sacral alae and a horizontal fracture line passing through the sacral body may be visible on a CT scan. 

The characteristic result on isotope bone scintigraphy or PET is the well-known “H sign,” observed in up to 40% of the cases, indicating enhanced radiotracer uptake along the fracture sites [101]. CT is more sensitive than radiography but less accurate than MRI in detecting SI joint damage [102]. Unless MRI is contraindicated, CT is generally not advised to establish the diagnosis of sacroiliitis [103]. However, when used, a low-radiation CT is sufficient [104].

The most common finding on STIR sequences for acute insufficiency fractures on MRI is florid BME. In later stages, both sacral alae show irregular vertical hypointense fracture lines surrounded by BME. The diagnosis of insufficiency fractures is supported by the presence of additional fractures in characteristic locations such as the pubic rami.

It is evident that most individuals with inflammatory sacroiliitis do not share the same demographic profile as those with sacral insufficiency fractures. Therefore, the suspicion of sacral insufficiency fractures should be raised by the bilateral and rather symmetrical distribution of the BME, occasionally with the previously mentioned pattern, the presence of fracture lines, the lack of structural alterations in the SI joints, and the patient’s age.

## 9. Neoplasia or Metastasis

**Figure 16:**
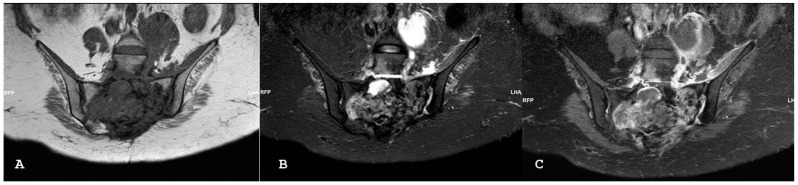
Coronal T1-weighted (**A**) and coronal short tau inversion recovery (**B**) magnetic resonance imaging sequences demonstrate destructive soft tissue mass centered in the sacral bone, consistent with a metastatic lesion, in a patient with uterine sarcoma. Coronal T1-weighted fat sat sequence with intravenous gadolinium magnetic resonance imaging sequence (**C**) demonstrates the extent of abnormal soft tissue enhancement and abscess formation within the left ileo-psoas muscle.

**Figure 17:**
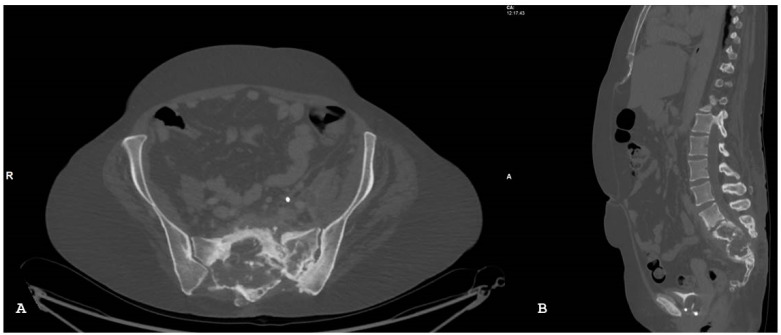
Same patient of Figure 16; axial (**A**) and sagittal (**B**) computed tomography images with bone windows show a destructive soft tissue lesion within the sacrum.

The sacrum is a rare but notable location for primary tumors and metastatic diseases. Metastatic sacral disease accounts for 1% to 7% of metastatic spine diseases [105]. While rectal carcinoma can directly infiltrate the sacrum, most sacral metastatic tumors arise from tumor cells that spread hematogenously [106]. The Batson venous plexus is a common route for metastases, particularly in the thoracic spine [107]. Primary sacral tumors are less common than myeloma, lymphoma, and metastases [108]. Sacral metastases are often discovered only after they have extended beyond the osseous boundaries, affecting the sacral nerves and nearby organs [109] (Figure 16 and Figure 17).

Sacral nerve root compression and pathological fractures are the major causes of pain in patients [101]. Moreover, bowel or bladder incontinence may result from sacral nerve root compression. A history of malignancy increases the risk of thromboembolism, a risk further exacerbated by decreased ambulation linked to radicular symptoms and/or pathological fractures [110]. The sacrum can also be the primary site for various cancers, including bone, neurogenic, and germ cell tumors [108]. However, the most frequent and distinctive primary tumors in this area are osteoblastomas, giant cell tumors, osteomas, and osteoid osteomas [111]. These tumors typically suggest an extension of soft or osseous tissue and infrequently mimic sacroiliitis.

Spine giant cell tumors are rare, constituting only 3–7% of all giant cell tumors, with the majority arising in the sacrum. Unlike chordomas, which are core lesions, sacral giant cell tumors often have an eccentric shape and either abut or span the SI joint. Most tumors are found in women, with a 2:1 female–male ratio, and the most affected patients are between 15 and 40 years old [112]. The most frequent presenting symptoms are pain and neurological impairments. Within a spindle cell stroma, osteoclastic giant cells make up a giant cell tumor, often accompanied by fibrotic and hemorrhagic regions. Less than 2% of individuals experience spontaneous malignant transformation, which typically occurs following radiation therapy [113]. 

Giant cell tumors are unique lesions that cause bone lysis and destruction without septations or matrix calcifications. Radiography remains the initial imaging modality used for diagnosis. However, given the difficulty in assessing the sacrum on radiographs, these may often be insufficient [114]. Furthermore, even in patients with confirmed sacral disease, radiographic examination has been shown to be inaccurate. Instead, the loss of the sacral arcuate line is a more strongly correlated marker of metastatic disease, and clinicians should pay particular attention to this finding [115].

Approximately 10% of osteoid osteomas occur in the spine, with only 2% located in the sacrum [116]. Males are affected two to three times more frequently than females, typically between the ages of 10 and 20. Patients often experience nocturnal pain, which is alleviated by nonsteroidal anti-inflammatory medications. Most osteoid osteomas originate in the articular process of the S1 vertebra. These tumors present as a radiolucent lesion less than 2 cm in diameter, surrounded by notable perifocal sclerosis. Central calcification within the osteolytic nidus is often observed. 

CT imaging is particularly helpful in diagnosing and detecting spinal osteoid osteomas. While the nidus typically exhibits low signal intensity on T1-weighted images and intermediate to high signal intensity on T2-weighted images, the efficacy of MRI in identifying the nidus remains uncertain. Some patients may exhibit soft-tissue edema, BME, or surrounding sclerosis, which can obscure the visibility of the nidus.

Osteoblastomas are uncommon lesions, accounting for only 1% to 2% of primary bone tumors. Approximately 40% of all osteoblastomas occur in the spine, with the sacrum representing 17% of these spinal cases. There is a slight male predominance, with a mean age of 20 years at presentation. The most frequent symptoms include pain, scoliosis, and neurologic impairments. Spinal osteoblastomas most commonly affect the posterior vertebral elements.

Due to their similar clinical and histologic features, osteoid osteoma and osteoblastoma may be considered distinct forms of the same underlying disease. Osteoblastomas typically present as a lytic defect greater than 1.5 cm in diameter, surrounded by a sclerotic ring. When calcifications are present, they are typically numerous. Osteoblastomas that extend into adjacent soft tissue and cause cortical damage can appear aggressive in certain patients. Signal intensity patterns on MRI are often vague. The typical finding of peripheral edema in soft tissue and bone marrow, known as the flare phenomenon, indicates an inflammatory reaction to the lesion.

Chordomas are very rare, accounting for only 2% to 4% of all primary malignant bone tumors. However, they are the most frequent primary malignant sacral tumor, comprising 20% to 34% of the cases [117].

**Figure 18:**
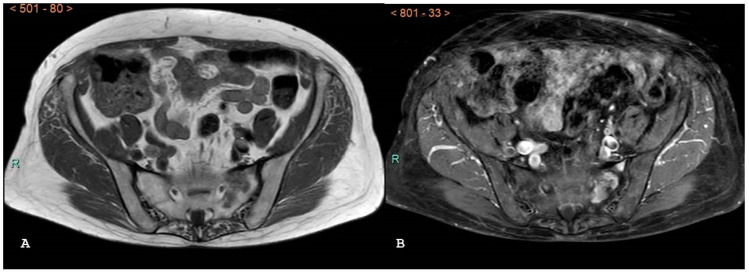
Axial T1-weighted (**A**) and axial T1 fat-saturated after gadolinium contrast (**B**) magnetic resonance imaging sequences demonstrate a metastatic lesion in the left sacral ala, adjacent to the sacroiliac joint, in a 70-yr-old woman with myeloma. The lesion appears hypointense in the T1-weighted sequence and has inhomogeneous contrastographic enhancement prevalent at the periphery related to the localization of disease in a nonactive phase.

Intraosseous notochordal remnants give rise to this tumor. The sacrococcygeal area, specifically the fourth and fifth sacral segments, is the genesis of about 50% of all chordomas. Merely 35% of chordomas are in the sphenooccipital area, whereas 15% are found in the spine above the sacrum. Patients are 50 years old on average, with men suffering twice as often as women. Large masses are frequently found in chordoma tumors, which grow slowly. Common chordomas are characterized by an abundance of intracellular and extracellular mucin as well as clear cells with intracytoplasmic vacuoles (physaliphorous cells). In chondroid or osteoid elements, the mucinous matrix is substituted in atypical or dedifferentiated chordomas. Sacral chordomas appear as big, lytic lesions centered in the midline with a soft-tissue mass associated with them on CT scans. Thirty to seventy percent of patients have calculi. When compared to skeletal muscle, chordomas are usually hyperintense on T2-weighted scans and iso- or slightly hypointense on T1-weighted imaging. The intratumoral mucin buildup is correlated with these chordomas. In total, 10% of people over 80 have Paget’s disease, which is a prevalent condition. Multiple myeloma, lymphoma, sarcoma, metastases, and giant cell tumors are among the neoplasms linked to Paget’s disease [118]. Less than 1% of individuals have been found to experience sarcomatous degeneration, an uncommon clinical consequence. The most common cause of this deterioration is osteosarcoma, which is followed by chondrosarcoma and malignant fibrous histiocytoma. Sarcomas are more common in older people with Paget’s disease and in those whit polyostotic disease. The skeletal distribution of the disease is reflected in the most prevalent sites, which are the femur, sacrum, and pelvis. With penetrating lysis, cortical destruction, and big accompanying soft-tissue masses, the imaging look typically implies an aggressive tumor with a high degree of anaplasia that is typically discovered on pathologic investigation. A distinctive calcification of the osseous matrix or chondroid may occasionally be seen in osteosarcomas and chondrosarcomas, respectively. Malignant bone marrow plasma cells proliferate monoclonals in multiple myeloma. The unifocal tumoral variant of multiple myeloma, known as plasmacytoma, typically has a better prognosis than multiple myeloma. Of all vertebral tumors, multiple myeloma accounts for 45% of the cases. The development of multicentric illness frequently occurs before plasmacytoma. Multiple myeloma most commonly affects the axial skeleton. When compared to healthy marrow on T1-weighted MRI scans, plasmacytomas and myeloma lesions are hypointense, while on T2-weighted imaging, they are hyperintense (Figure 18).

## 10. Pearls and Pitfalls of SI Joint Imaging

When imaging the SI joints, radiologists should know a few useful pointers, especially when using MRI. While MRI is quite sensitive in identifying SpA, BME can also be identified in healthy individuals and is more common in those over 30 [119,120]. The physiologic subchondral BME that occurs in children and adolescents can sometimes persist into early adulthood and may be a mistake in the diagnosis of sacroiliitis [119]. However, the extent is limited, and deep and/or intense BME is relatively rare and most commonly occurs in the superior portion of the sacrum (both anterior and posterior), followed by the inferior ilium, and is primarily caused by strain [119]. Because mechanical back pain can also cause isolated alterations in periarticular BME, the overall specificity of MRI for SpA is reduced to 88%. Non-specific observations such as focal or patchy fat depositions inside the bone marrow are becoming more common in older, healthy persons and in a degenerative setting; nevertheless, they do not appear in subchondral locations [119,120]. Lesions resembling erosion or erosion itself may also be typical. However, none of the healthy individuals between the ages of 20 and 29 met the suggested structural criteria for axial SpA sacroiliitis using cut-off values for erosions and fat metaplasia with a 95% specificity for axial SpA (erosions in three quadrants, or erosions and/or fat metaplasia in five quadrants) [38]. The prevalence of erosions also increased with age. There is a dearth of information on sclerosis, osteophytes, and changes in joint space at normal SI joint by MRI [121,122,123]. Because fracture lines may not be visible in the early stages and florid STIR signal hyperintensity may be misinterpreted for inflammatory or metastatic illness, acute insufficiency fractures can be deceptive on MRI. Additionally, readers must exercise caution to avoid mistaking artifacts like periarticular arteries or inhomogeneous fat suppression for edema or augmentation in the bone marrow. Finally, it is critical to keep in mind that each instance of unilateral sacroiliitis should be diagnosed with an infection, and that early soft tissue alterations should be detected using contrast-enhanced MRI.

## 11. Conclusions

The most common causes of SI pain are inflammatory or infectious disorders; however, it can also be caused by benign or malignant reasons, which are less common. SI discomfort can be caused by a wide number of things. To obtain a correct diagnosis of sacroiliitis, especially in cases of doubt, a multidisciplinary approach by rheumatologists and radiologists is necessary to ensure that clinical, laboratory, and imaging findings support the diagnosis.

Both clinicians and radiologists should be aware of the benefits that each imaging modality offers, and imaging studies should be customized to the specific clinical presentation of each individual patient. When it comes to the detection of sacroiliitis and other noninflammatory mimickers, cross-sectional modalities are preferable and MRI is the gold standard imaging technique due to its sensitivity in detecting early and inflammatory changes and can facilitate early diagnosis and treatment.

## Figures and Tables

**Table 1:** Inflammatory back pain (IBP) parameters according to Assessments in SpondyloArthritis international Society experts [10].

Age at onset < 40 year
2. Insidious onset
3. Improvement with exercise
4. No improvement with rest
5. Pain at night (with improvement upon getting up)
Performance: sensitivity 77.0% and specificity 91.7% if at least four out of five parameters are present. Note that sensitivity and specificity refer to the presence of IBP, not to diagnosis.

**Table 2:** Assessments of SpondyloArthritis international Society MRI Working Group consensus definitions for MRI lesions in the sacroiliac joint of patients with spondyloarthritis [19].

**Overarching principles**
1. When interpreting medical studies of the SIJ in SpA for diagnostic or classification purposes, all the available images for that modality should be reviewed at the same time as different slice orientations or sequences may provide additional information that is important for the correct interpretation of the findings. MR images that illustrate different features of sacroiliitis compatible with (or highly suggestive of) SpA, such as active disease and structural damage, should be simultaneously reviewed and interpreted in the context of all findings.
2. Many artifacts occur on MRI of the SIJ and when a feature of uncertain significance is seen in one orientation then, if possible, the feature should be verified in a second orientation. This may be more important for imaging studies used for diagnostic or classification purposes.
3. The SIJ lesion(s) must be clearly present and located in a typical anatomical location, and its appearance must be highly suggestive of SpA. The presence of any small solitary lesion should be interpreted with caution. It is rare for a lesion to be “clearly present” if small and solitary and it is expected that relevant lesions will be either multiple or seen on multiple images (slices, sequences, orientation). If a lesion appears to be present but it is hard to determine whether the lesion is “highly suggestive of SpA”, then the decision may be influenced by the presence of other concomitant lesions.
4. The visual interpretation of an MRI scan of the SIJ should be performed objectively. In the research setting, the interpretation will usually be performed in the absence of patient data. But, the clinician should interpret the MRI report in the total context of the demographic, clinical, and laboratory information from the patient, and although the MRI of the SIJ may be reported as suggestive of SpA, the final decision can still be that the patient has no SpA. Other conditions of the SIJ such as fracture, osteoarthritis, sepsis, trauma, neoplasia, and artifacts may resemble lesions observed on MRI in patients with SpA.
**MRI sacroiliac joint lesion definitions indicating signs of activity**
These observations are made on MRI sequences that are sensitive for the detection of disease activity such as T2-weighted (T2W) sequences with fat suppression (FS) that are sensitive for free water (e.g., short tau inversion recovery (STIR)), or T1W sequences with fat suppression that are sensitive for contrast enhancement such as T1WFS post gadolinium (Gd).
ASAS definition of positive MRI for the classification of SpA: MRI evidence of bone marrow inflammation must be present and the features required for the definition of active sacroiliitis on MRI are: bone marrow edema (BME) on a T2W sequence sensitive for free water (e.g., STIR, T2FS) or bone marrow contrast enhancement on a T1W sequence (e.g., T1FS post-Gd). BME is depicted as a hyperintense signal on STIR images and usually as a hypointense signal on T1 images. A hyperintense signal on contrast-enhanced, T1-weighted, fat-saturated images (T1 post-Gd) reflects increased vascularization and is referred to as osteitis. The sacral interforaminal bone marrow signal forms the reference for the assignment of normal signal in the bone marrow; inflammation must be clearly present and located in a typical anatomical area (subchondral bone); MRI appearance must be highly suggestive of SpA.
2. Capsulitis: increased signal on STIR and/or T1FS post-Gd, which is observed at the perimeter of the joint (anterior or posterior on axial images, cranial or caudal on semicoronal images).
3. Joint space enhancement: increased signal on contrast-enhanced images in the joint space of the cartilaginous portion of the SIJ.
4. Inflammation at the site of erosion: increased signal on STIR and/or T1FS post-Gd at the site of erosion.
5. Enthesitis: increased signal in bone marrow and/or soft tissue on STIR and/or T1FS post-Gd at sites where ligaments and tendons attach to bone, but not including the inter-osseous ligaments of the sacroiliac joint.
6. Joint space fluid: Bright signal in the joint space on STIR images equivalent to cerebrospinal fluid.
**MRI sacroiliac joint lesion definitions indicating signs of structural change**
These observations are made on MRI sequences that are sensitive to the detection of structural change. Most of the observations can only be seen clearly on sequences sensitive to fat signal, specifically T1W spin echo without fat suppression.
Erosion: a defect in subchondral bone associated with full thickness loss of the dark appearance of the subchondral cortex at its expected location, with loss of signal on a T1W non-fat-suppressed sequence compared to the normal bright appearance of adjacent bone marrow.
2. Fat lesion (also known as fat metaplasia): bright signal seen on a T1W non-fat-suppressed sequence that is brighter than normal bone marrow, which meets the following requirements: Homogeneously bright; Located in a typical anatomical area (subchondral bone); Sharply defined along its non-articular border with normal bone marrow.
3. Fat metaplasia in an erosion cavity (also known as “backfill”): bright signal on a T1-weighted sequence in a typical location for an erosion or confluent erosions, with signal intensity greater than normal bone marrow, which meets the following requirements: Associated with complete loss of the dark appearance of the subchondral cortex at its expected location; Clearly demarcated from adjacent bone marrow by an irregular band of dark signal reflecting sclerosis at the border of the original erosion.
4. Sclerosis: very low signal on all sequences located in a typical anatomical area (subchondral bone).
5. Ankylosis: abnormal bright signal on a T1W non-fat-suppressed sequence with similar signal intensity to bone marrow, which is in the expected location of the sacroiliac joint space and bridges the joint so that there is continuity of bone marrow signal between the ilium and sacrum. It is associated with full-thickness loss of the dark appearance of the subchondral cortex on both sides of the joint.
6. Bone bud: abnormal bright signal on a T1W non-fat-suppressed sequence with similar signal intensity to bone marrow, which is in the expected location of the sacroiliac joint space but does not bridge the joint so that it is continuous with the subchondral bone of either the ilium or the sacrum but not both. It is associated with full thickness loss of the dark appearance of the subchondral cortex on the corresponding side of the joint, at its expected 7. location.

**Table 3:** Assessments of SpondyloArthritis international Society classification criteria for axial spondyloarthritis [20,21].

Chronic Back Pain (≥ 3 months) and Age at Onset < 45 Years			
Plus			
Sacroileitis on imaging ≥1 SpA feature	or		HLA-B27 positivity≥2 other SpA feature
SpA features			
Inflammatory back pain Peripheral arthritis Dactylitis Enthesitis (heel) Family history of SpA		Good response to NSAIDs Uveitis Psoriasis Crohn’s/Colitis HLA-B27 Elevated CRP	

Abbreviations: HLA = human leukocyte antigen; SpA = spondyloarthritis; NSAIDs = non-steroidal anti-inflammatory drugs; CRP = C-reactive protein.

## Data Availability

Not applicable.

## References

[1] Vereecke E., Diekhoff T., Eshed I., Herregods N., Morbée L., Jaremko J.L., Jans L. (2024). ESR Essentials: Imaging of sacroiliitis-practice recommendations by ESSR. Eur. Radiol..

[2] Van der Linden S., Valkenburg H.A., Cats A. (1984). Evaluation of diagnostic criteria for ankylosing spondylitis. A proposal for modification of the New York criteria. Arthritis Rheum..

[3] Carneiro B.C., Rizzetto T.A., Silva F.D., da Cruz I.A.N., Guimarães J.B., Ormond Filho A.G., Nico M.A.C. (2022). Sacroiliac joint beyond sacroiliitis-further insights and old concepts on magnetic resonance imaging. Skelet. Radiol..

[4] Özgen A. (2017). The Value of the T2-Weighted Multipoint Dixon Sequence in MRI of Sacroiliac Joints for the Diagnosis of Active and Chronic Sacroiliitis. Am. J. Roentgenol..

[5] Al-Mnayyis A., Obeidat S., Badr A., Jouryyeh B., Azzam S., Al Bibi H., Al-Gwairy Y., Al Sharie S., Varrassi G. (2024). Radiological Insights into Sacroiliitis: A Narrative Review. Clin. Pract..

[6] Li X., Wang J., Li P., Zhuang S., Jiang S., Liu W. (2024). Accuracy of dual-energy computed tomography for bone marrow edema in the sacroiliac joint: A systematic review and meta-analysis. Medicine.

[7] Wu H., Zhang G., Shi L., Li X., Chen M., Huang X., Cao X., Tan S., Cui Y., Liang C. (2019). Axial Spondyloarthritis: Dual-Energy Virtual Noncalcium CT in the Detection of Bone Marrow Edema in the Sacroiliac Joints. Radiology.

[8] Carotti M., Benfaremo D., Di Carlo M., Ceccarelli L., Luchetti M.M., Piccinni P., Giovagnoni A., Salaffi F. (2021). Dual-energy computed tomography for the detection of sacroiliac joints bone marrow oedema in patients with axial spondyloarthritis. Clin. Exp. Rheumatol..

[9] Kandagaddala M., Sathyakumar K., Mathew A.J., Regi S.S., Yadav B., David K., Danda D. (2024). MRI predictors of infectious etiology in patients with unilateral sacroiliitis. Int. J. Rheum. Dis..

[10] Sieper J., Rudwaleit M., Baraliakos X., Brandt J., Braun J., Burgos-Vargas R., Dougados M., Hermann K.G., Landewé R., Maksymowych W. (2009). The Assessment of SpondyloArthritis international Society (ASAS) handbook: A guide to assess spondyloarthritis. Ann. Rheum. Dis..

[11] Muche B., Bollow M., François R.J., Sieper J., Hamm B., Braun J. (2003). Anatomic structures involved in early- and late-stage sacroiliitis in spondylarthritis: A detailed analysis by contrast-enhanced magnetic resonance imaging. Arthritis Rheum..

[12] Smolen J.S., Schöls M., Braun J., Dougados M., FitzGerald O., Gladman D.D., Kavanaugh A., Landewé R., Mease P., Sieper J. (2018). Treating axial spondyloarthritis and peripheral spondyloarthritis, especially psoriatic arthritis, to target: 2017 update of recommendations by an international task force. Ann. Rheum. Dis..

[13] Da Silva A.B., Lourenço M.H., Ramiro S., Falzon L., Cunha-Branco J., van der Heijde D., Landewé R., Sepriano A. (2024). Performance of clinical, laboratory and imaging features for diagnosing spondyloarthritis-a systematic literature review and meta-analysis. Rheumatology.

[14] Poddubnyy D. (2020). Classification vs diagnostic criteria: The challenge of diagnosing axial spondyloarthritis. Rheumatology.

[15] Feldtkeller E., Bruckel J., Khan M.A. (2000). Scientific contributions of ankylosing spondylitis patient advocacy groups. Curr. Opin. Rheumatol..

[16] Masson Behar V., Dougados M., Etcheto A., Kreis S., Fabre S., Hudry C., Dadoun S., Rein C., Pertuiset E., Fautrel B. (2017). Diagnostic delay in axial spondyloarthritis: A cross-sectional study of 432 patients. Jt. Bone Spine.

[17] Van den Berg R., Lenczner G., Feydy A., van der Heijde D., Reijnierse M., Saraux A., Rahmouni A., Dougados M., Claudepierre P. (2014). Agreement between clinical practice and trained central reading in reading of sacroiliac joints on plain pelvic radiographs. Results from the DESIR cohort. Arthritis Rheumatol..

[18] Van Tubergen A., Heuft-Dorenbosch L., Schulpen G., Landewé R., Wijers R., van der Heijde D., van Engelshoven J., van der Linden S. (2003). Radiographic assessment of sacroiliitis by radiologists and rheumatologists: Does training improve quality?. Ann. Rheum. Dis..

[19] Lambert R.G.W., Baraliakos X., A Bernard S., A Carrino J., Diekhoff T., Eshed I., A Hermann K.G., Herregods N., Jaremko J., Jans L.B. (2024). Development of international consensus on a standardised image acquisition protocol for diagnostic evaluation of the sacroiliac joints by MRI: An ASAS-SPARTAN collaboration. Ann. Rheum. Dis..

[20] Rudwaleit M., Jurik A.G., Hermann K.G., Landewé R., van der Heijde D., Baraliakos X., Marzo-Ortega H., Ostergaard M., Braun J., Sieper J. (2009). Defining active sacroiliitis on magnetic resonance imaging (MRI) for classification of axial spondyloarthritis: A consensual approach by the ASAS/OMERACT MRI group. Ann. Rheum. Dis..

[21] Rudwaleit M., Landewe R., van der Heijde D., Listing J., Brandt J., Braun J., Burgos-Vargas R., Collantes-Estevez E., Davis J., Dijkmans B. (2009). The development of Assessment of SpondyloArthritis international Society classification criteria for axial spondyloarthritis (part I): Classification of paper patients by expert opinion including uncertainty appraisal. Ann. Rheum. Dis..

[22] Moltó A., Paternotte S., van der Heijde D., Claudepierre P., Rudwaleit M., Dougados M. (2015). Evaluation of the validity of the different arms of the ASAS set of criteria for axial spondyloarthritis and description of the different imaging abnormalities suggestive of spondyloarthritis: Data from the DESIR cohort. Ann. Rheum. Dis..

[23] Sepriano A., Rubio R., Ramiro S., Landewé R., van der Heijde D. (2017). Performance of the ASAS classification criteria for axial and peripheral spondyloarthritis: A systematic literature review and meta-analysis. Ann. Rheum. Dis..

[24] Gondim Teixeira P.A., Bravetti M., Hossu G., Lecocq S., Petit D., Loeuille D., Blum A. (2017). Protocol optimization of sacroiliac joint MR Imaging at 3 Tesla: Impact of coil design and motion resistant sequences on image quality. Diagn. Interv. Imaging.

[25] Maksymowych W.P., Wichuk S., Chiowchanwisawakit P., Lambert R.G., Pedersen S.J. (2017). Fat metaplasia on MRI of the sacroiliac joints increases the propensity for disease progression in the spine of patients with spondyloarthritis. RMD Open.

[26] de Hooge M., Pialat J.B., Reijnierse M., van der Heijde D., Claudepierre P., Saraux A., Dougados M., Feydy A. (2017). Assessment of typical SpA lesions on MRI of the spine: Do local readers and central readers agree in the DESIR-cohort at baseline?. Clin. Rheumatol..

[27] Bakker P.A., van den Berg R., Lenczner G., Thévenin F., Reijnierse M., Claudepierre P., Wendling D., Dougados M., van der Heijde D. (2017). Can we use structural lesions seen on MRI of the sacroiliac joints reliably for the classification of patients according to the ASAS axial spondyloarthritis criteria? Data from the DESIR cohort. Ann. Rheum. Dis..

[28] Dougados M., Sepriano A., Molto A., van Lunteren M., Ramiro S., de Hooge M., van den Berg R., Navarro Compan V., Demattei C., Landewé R. (2017). Sacroiliac radiographic progression in recent onset axial spondyloarthritis: The 5-year data of the DESIR cohort. Ann. Rheum. Dis..

[29] Neuenschwander R., Hebeisen M., Micheroli R., Bürki K., Exer P., Niedermann K., Nissen M.J., Scherer A., Ciurea A. (2020). Differences between men and women with nonradiographic axial spondyloarthritis: Clinical characteristics and treatment effectiveness in a real-life prospective cohort. Arthritis Res. Ther..

[30] Blachier M., Canouï-Poitrine F., Dougados M., Lethuaut A., Fautrel B., Ferkal S., Le Corvoisier P., Farrenq V., Poulain C., Ghaleh B. (2013). Factors associated with radiographic lesions in early axial spondyloarthritis. Results from the DESIR cohort. Rheumatology.

[31] Heuft-Dorenbosch L., Weijers R., Landewé R., van der Linden S., van der Heijde D. (2006). Magnetic resonance imaging changes of sacroiliac joints in patients with recent-onset inflammatory back pain: Inter-reader reliability and prevalence of abnormalities. Arthritis Res. Ther..

[32] van den Berg R., Lenczner G., Thévenin F., Claudepierre P., Feydy A., Reijnierse M., Saraux A., Rahmouni A., Dougados M., van der Heijde D. (2015). Classification of axial SpA based on positive imaging (radiographs and/or MRI of the sacroiliac joints) by local rheumatologists or radiologists versus central trained readers in the DESIR cohort. Ann. Rheum. Dis..

[33] Weber U., Lambert R.G., Østergaard M., Hodler J., Pedersen S.J., Maksymowych W.P. (2010). The diagnostic utility of magnetic resonance imaging in spondylarthritis: An international multicenter evaluation of one hundred eighty-seven subjects. Arthritis Rheumatol..

[34] Arnbak B., Grethe Jurik A., Hørslev-Petersen K., Hendricks O., Hermansen L.T., Loft A.G., Østergaard M., Pedersen S.J., Zejden A., Egund N. (2016). Associations between Spondyloarthritis Features and Magnetic Resonance Imaging Findings: A Cross-Sectional Analysis of 1,020 Patients with Persistent Low Back Pain. Arthritis Rheumatol..

[35] Deodhar A. (2016). Sacroiliac Joint Magnetic Resonance Imaging in the Diagnosis of Axial Spondyloarthritis: “A Tiny Bit of White on Two Consecutive Slices” May Be Objective, but Not Specific. Arthritis Rheumatol..

[36] De Winter J., de Hooge M., van de Sande M., de Jong H., van Hoeven L., de Koning A., Berg I.J., Ramonda R., Baeten D., van der Heijde D. (2018). Magnetic Resonance Imaging of the Sacroiliac Joints Indicating Sacroiliitis According to the Assessment of SpondyloArthritis international Society Definition in Healthy Individuals, Runners, and Women with Postpartum Back Pain. Arthritis Rheumatol..

[37] Maksymowych W.P., Inman R.D., Salonen D., Dhillon S.S., Williams M., Stone M., Conner-Spady B., Palsat J., Lambert R.G. (2005). Spondyloarthritis research Consortium of Canada magnetic resonance imaging index for assessment of sacroiliac joint inflammation in ankylosing spondylitis. Arthritis Rheumatol..

[38] Herregods N., Anisau A., Schiettecatte E., Vereecke E., Morbée L., Laloo F., Jaremko J.L., Jans L. (2023). MRI in pediatric sacroiliitis, what radiologists should know. Pediatr. Radiol..

[39] Kucera T., Brtkova J., Sponer P., Ryskova L., Popper E., Frank M., Kucerova M. (2015). Pyogenic sacroiliitis: Diagnosis, management and clinical outcome. Skelet. Radiol..

[40] Barnes M., Bush C., Jones J. (2019). Pyogenic sacroiliitis: A rare complication of inflammatory bowel disease. Am. J. Emerg. Med..

[41] Cosma S., Borella F., Carosso A., Ingala A., Fassio F., Robba T., Maina A., Bertero L., Benedetto C. (2019). Osteomyelitis of the pubic symphysis caused by methicillin-resistant *Staphylococcus aureus* after vaginal delivery: A case report and literature review. BMC Infect. Dis..

[42] Cardoso L., Alves P., Santos F., Ross J.J. (2017). Septic arthritis of the pubic symphysis. BMJ Case Rep..

[43] Stürzenbecher A., Braun J., Paris S., Biedermann T., Hamm B., Bollow M. (2000). MR imaging of septic sacroiliitis. Skelet. Radiol..

[44] Wang Y.Y., Zhao Z., Zhang J.L., Huang F. (2020). Clinical and imaging characteristics of 110 patients with infectious sacroiliitis. Zhonghua Nei Ke Za Zhi.

[45] Sondag M., Gete K., Verhoeven F., Aubry S., Prati C., Wendling D. (2019). Analysis of the early signs of septic sacroiliitis on computed tomography. Eur. J. Rheumatol..

[46] Hermet M., Minichiello E., Flipo R.M., Dubost J.J., Allanore Y., Ziza J.M., Gaudin P., Thomas T., Dernis E., Glace B. (2012). Infectious sacroiliitis: A retrospective, multicentre study of 39 adults. BMC Infect. Dis..

[47] Novaes F.S., Shimo A.K., Lopes M.H. (2006). Lombalgia na gestação. Low back pain during gestation. Rev. Lat. Am. Enfermagem..

[48] Shi C., Zou Q., Wei H. (2024). The association of back pain with physical inactivity and hypothyroidism in pregnant women. J. Back Musculoskelet. Rehabil..

[49] De Sousa V.P.S., Cury A., Eufrásio L.S., de Sousa S.E.S., Coe C.B., de Souza Ramalho Viana E. (2019). The influence of gestational trimester, physical activity practice and weight gain on the low back and pelvic pain intensity in low risk pregnant women. J. Back Musculoskelet. Rehabil..

[50] Albert H.B., Godskesen M., Westergaard J.G. (2002). Incidence of four syndromes of pregnancy-related pelvic joint pain. Spine.

[51] Albert H., Godskesen M., Westergaard J. (2001). Prognosis in four syndromes of pregnancy-related pelvic pain. Acta Obs. Gynecol. Scand..

[52] Eshed I., Miloh-Raz H., Dulitzki M., Lidar Z., Aharoni D., Liberman B., Lidar M. (2015). Peripartum changes of the sacroiliac joints on MRI: Increasing mechanical load correlating with signs of edema and inflammation kindling spondyloarthropathy in the genetically prone. Clin. Rheumatol..

[53] Hamilton L., Macgregor A., Warmington V., Pinch E., Gaffney K. (2014). The prevalence of inflammatory back pain in a UK primary care population. Rheumatology.

[54] Reveille J.D., Weisman M.H. (2013). The epidemiology of back pain, axial spondyloarthritis and HLA-B27 in the United States. Am. J. Med. Sci..

[55] Stolwijk C., Boonen A., van Tubergen A., Reveille J.D. (2012). Epidemiology of spondyloarthritis. Rheum. Dis. Clin. N. Am..

[56] Ferlin A., De Toni L., Sandri M., Foresta C. (2017). Relaxin and insulin-like peptide 3 in the musculoskeletal system: From bench to bedside. Br. J. Pharmacol..

[57] Daneau C., Houle M., Pasquier M., Ruchat S.M., Descarreaux M. (2021). Association between Pregnancy-Related Hormones and Lumbopelvic Pain Characteristics in Pregnant Women: A Scoping Review. J. Manip. Physiol. Ther..

[58] Fiani B., Sekhon M., Doan T., Bowers B., Covarrubias C., Barthelmass M., De Stefano F., Kondilis A. (2021). Sacroiliac Joint and Pelvic Dysfunction Due to Symphysiolysis in Postpartum Women. Cureus.

[59] Yan C.X., Vautour L., Martin M.H. (2016). Postpartum sacral insufficiency fractures. Skelet. Radiol..

[60] Germann C., Kroismayr D., Brunner F., Pfirrmann C.W.A., Sutter R., Zubler V. (2021). Influence of pregnancy/childbirth on long-term bone marrow edema and subchondral sclerosis of sacroiliac joints. Skelet. Radiol..

[61] Agten C.A., Zubler V., Zanetti M., Binkert C.A., Kolokythas O., Prentl E., Buck F.M., Pfirrmann C.W.A. (2018). Postpartum Bone Marrow Edema at the Sacroiliac Joints May Mimic Sacroiliitis of Axial Spondyloarthritis on MRI. Am. J. Roentgenol..

[62] Wurdinger S., Humbsch K., Reichenbach J.R., Peiker G., Seewald H.J., Kaiser W.A. (2002). MRI of the pelvic ring joints postpartum: Normal and pathological findings. J. Magn. Reson. Imaging.

[63] Hermann K.G., Halle H., Reisshauer A., Schink T., Vsianska L., Mühler M.R., Lembcke A., Hamm B., Bollow M. (2007). Peripartale Veränderungen des Beckenringes: Wie sinnvoll ist die Magnetresonanztomografie? Peripartum changes of the pelvic ring: Usefulness of magnetic resonance imaging. Rofo.

[64] Hoballah A., Lukas C., Leplat C., Taourel P., Pialat J.B., Sans N., Ramos-Pascual S., Cyteval C. (2020). MRI of sacroiliac joints for the diagnosis of axial SpA: Prevalence of inflammatory and structural lesions in nulliparous, early postpartum and late postpartum women. Ann. Rheum. Dis..

[65] Berthelot J.M., le Goff B., Maugars Y., Laredo J.D. (2016). Sacroiliac joint edema by MRI: Far more often mechanical than inflammatory?. Jt. Bone Spine.

[66] La Paglia E., Zawaideh J.P., Lucii G., Mazzei M.A. (2019). MRI of the axial skeleton: Differentiating non-inflammatory diseases and axial spondyloarthritis: A review of current concepts and applications: Special issue on “musculoskeletal imaging of the inflammatory and degenerative joints: Current status and perspectives”. Radiol. Med..

[67] Egbuchiem H., Onyechi N., Okwori O.F., Oshobe A. (2023). *Osteitis condensans* Ilii: An Uncommon Cause of Back Pain Masquerading as an Inflammatory Spondyloarthropathy. Cureus.

[68] Parperis K., Psarelis S., Nikiphorou E. (2020). *Osteitis condensans* ilii: Current knowledge and diagnostic approach. Rheumatol. Int..

[69] Dharmshaktu G.S., Dharmshaktu I.S. (2023). *Osteitis condensans* Ilii: A Mini Review. Ann. Rheumatol. Autoimmun..

[70] Jenks K., Meikle G., Gray A., Stebbings S. (2009). *Osteitis condensans* ilii: A significant association with sacroiliac joint tenderness in women. Int. J. Rheum. Dis..

[71] Ma L., Gao Z., Zhong Y., Meng Q. (2018). *Osteitis condensans* ilii may demonstrate bone marrow edema on sacroiliac joint magnetic resonance imaging. Int. J. Rheum. Dis..

[72] Sicard J.A., Gally L., Haguenau J. (1926). *Osteites condensantes*, a etiologie inconnue. J. Radiol. D’electrol..

[73] Barsony T., Polgar F. (1928). Osteitis Condensans Ilei: Ein bisher nicht beschriebernes Krankheitsbild. Fortschr. Rontgen-Strahlen.

[74] Vadivelu R., Green T.P., Bhatt R. (2005). An uncommon cause of back pain in pregnancy. Postgrad. Med. J..

[75] Biswas S., Konala V.M., Adapa S., Amudala P., Naramala S. (2019). *Osteitis condensans* Ilii: An Uncommon Cause of Back Pain. Cureus.

[76] Eshed I., Lidar M. (2017). MRI Findings of the Sacroiliac Joints in Patients with Low Back Pain: Alternative Diagnosis to Inflammatory Sacroiliitis. Isr. Med. Assoc. J..

[77] Williams P.M., Byerly D.W. (2024). Osteitis Condensans Ilii. StatPearls.

[78] Testini V., Eusebi L., Guerra F.S., Manantrizio D., Guglielmi G. (2023). The promising role of DECT in the diagnosis of *Osteitis Condensans* Ilii: A case report. Acta Biomed..

[79] Pickhardt P.J., Pooler B.D., Lauder T., del Rio A.M., Bruce R.J., Binkley N. (2013). Opportunistic screening for osteoporosis using abdominal computed tomography scans obtained for other indications. Ann. Intern. Med..

[80] Fauny M., Verhoeven F., Allado E., Albuisson E., Pinzano A., Morizot C., Chary-Valckenaere I., Loeuille D. (2021). Relationship between spinal structural damage on radiography and bone fragility on CT in ankylosing spondylitis patients. Sci. Rep..

[81] Ulano A., Bredella M.A., Burke P., Chebib I., Simeone F.J., Huang A.J., Torriani M., Chang C.Y. (2016). Distinguishing Untreated Osteoblastic Metastases From Enostoses Using CT Attenuation Measurements. Am. J. Roentgenol..

[82] Sala F., Dapoto A., Morzenti C., Firetto M.C., Valle C., Tomasoni A., Sironi S. (2019). Bone islands incidentally detected on computed tomography: Frequency of enostosis and differentiation from untreated osteoblastic metastases based on CT attenuation value. Br. J. Radiol..

[83] Wong W.D., Shah S., Murray N., Walstra F., Khosa F., Nicolaou S. (2018). Advanced Musculoskeletal Applications of Dual-Energy Computed Tomography. Radiol. Clin. N. Am..

[84] Ishimura D., Morino T., Murakami Y., Yamaoka S., Kinoshita T., Takao M. (2023). Examining the Association between the Extent of Anterior Longitudinal Ligament Ossification Progression and Comorbidities in Diffuse Idiopathic Skeletal Hyperostosis. Cureus.

[85] Kim S.K., Choi B.R., Kim C.G., Chung S.H., Choe J.Y., Joo K.B., Bae S.C., Yoo D.H., Jun J.B. (2004). The prevalence of diffuse idiopathic skeletal hyperostosis in Korea. J. Rheumatol..

[86] Pariente E., Pini S.F., Olmos J.M., Fierro P., Landeras R., Ramos C., Martínez-Taboada V.M., Hernández J.L. (2023). Early stages of diffuse idiopathic skeletal hyperostosis (DISH) and chronic inflammation: The Camargo Cohort Study. Clin. Rheumatol..

[87] Pillai S., Littlejohn G. (2014). Metabolic factors in diffuse idiopathic skeletal hyperostosis—A review of clinical data. Open Rheumatol. J..

[88] Resnick D., Shaul S.R., Robins J.M. (1975). Diffuse idiopathic skeletal hyperostosis (DISH): Forestier’s disease with extraspinal manifestations. Radiology.

[89] Resnick D., Niwayama G. (1976). Radiographic and pathologic features of spinal involvement in diffuse idiopathic skeletal hyperostosis (DISH). Radiology.

[90] Littlejohn G.O., Urowitz M.B., Smythe H.A., Keystone E.C. (1981). Radiographic features of the hand in diffuse idiopathic skeletal hyperostosis (DISH): Comparison with normal subjects and acromegalic patients. Radiology.

[91] Utsinger P.D., Resnick D., Shapiro R. (1976). Diffuse skeletal abnormalities in Forestier disease. Arch. Intern. Med..

[92] Leibushor N., Slonimsky E., Aharoni D., Lidar M., Eshed I. (2017). CT Abnormalities in the Sacroiliac Joints of Patients with Diffuse Idiopathic Skeletal Hyperostosis. Am. J. Roentgenol..

[93] Ghossan R., Zebouni S.H., Farah T.Y., Fayad F. (2022). Diffuse Idiopathic Skeletal Hyperostosis and Ankylosing Spondylitis: A Challenging Case and Review of the Literature. J. Radiol. Case Rep..

[94] Olivieri I., D’Angelo S., Palazzi C., Padula A., Mader R., Khan M.A. (2009). Diffuse idiopathic skeletal hyperostosis: Differentiation from ankylosing spondylitis. Curr. Rheumatol. Rep..

[95] Yahara Y., Yasuda T., Kawaguchi Y., Suzuki K., Seki S., Kondo M., Makino H., Kamei K., Kanamori M., Kimura T. (2020). Sacroiliac joint variation associated with diffuse idiopathic skeletal hyperostosis. BMC Musculoskelet. Disord..

[96] Takahashi T., Yoshii T., Mori K., Kobayashi S., Inoue H., Tada K., Tamura N., Hirai T., Sugimura N., Nagoshi N. (2023). Comparison of radiological characteristics between diffuse idiopathic skeletal hyperostosis and ankylosing spondylitis: A multicenter study. Sci. Rep..

[97] Latourte A., Charlon S., Etcheto A., Feydy A., Allanore Y., Dougados M., Molto A. (2018). Imaging Findings Suggestive of Axial Spondyloarthritis in Diffuse Idiopathic Skeletal Hyperostosis. Arthritis Care Res..

[98] Bjelland E.K., Stuge B., Vangen S., Stray-Pedersen B., Eberhard-Gran M. (2013). Mode of delivery and persistence of pelvic girdle syndrome 6 months postpartum. Am. J. Obs. Gynecol..

[99] Brahme S.K., Cervilla V., Vint V., Cooper K., Kortman K., Resnick D. (1990). Magnetic resonance appearance of sacral insufficiency fractures. Skelet. Radiol..

[100] Donovan A., Schweitzer M.E., Rafii M., Lax A. (2009). Radiological features of superomedial iliac insufficiency fractures: A possible mimicker of metastatic disease. Skelet. Radiol..

[101] Finiels H., Finiels P.J., Jacquot J.M., Strubel D. (1997). Fractures du sacrum par insuffisance osseuse. Méta-analyse de 508 cas [Fractures of the sacrum caused by bone insufficiency. Meta-analysis of 508 cases]. Presse Med..

[102] Dydyk A.M., Forro S.D., Hanna A. (2020). Sacroiliac Joint Injury. StatPearls.

[103] Mandl P., Navarro-Compán V., Terslev L., Aegerter P., van der Heijde D., D’Agostino M.A., Baraliakos X., Pedersen S.J., Jurik A.G., Naredo E. (2015). European League Against Rheumatism (EULAR). EULAR recommendations for the use of imaging in the diagnosis and management of spondyloarthritis in clinical practice. Ann. Rheum. Dis..

[104] Diekhoff T., Hermann K.G., Greese J., Schwenke C., Poddubnyy D., Hamm B., Sieper J. (2017). Comparison of MRI with radiography for detecting structural lesions of the sacroiliac joint using CT as standard of reference: Results from the SIMACT study. Ann. Rheum. Dis..

[105] Quraishi N.A., Giannoulis K.E., Edwards K.L., Boszczyk B.M. (2012). Management of metastatic sacral tumours. Eur. Spine J..

[106] Kollender Y., Meller I., Bickels J., Flusser G., Issakov J., Merimsky O., Marouani N., Nirkin A., Weinbroum A.A. (2003). Role of adjuvant cryosurgery in intralesional treatment of sacral tumors. Cancer.

[107] Ziu E., Viswanathan V.K., Mesfin F.B. (2024). Spinal Metastasis. StatPearls.

[108] Sambri A., Fiore M., Giannini C., Pipola V., Zucchini R., Aparisi Gomez M.P., Aguiar P.M., Gasbarrini A., De Paolis M. (2022). Primary Tumors of the Sacrum: Imaging Findings. Curr. Med. Imaging.

[109] Safaee M.M., Carrera D.A., Chin C.T., Mashhood A., Eisenmenger L., Liang N.E., Lewis K.M., Chou D., Ames C.P., Weinstein P.R. (2020). Diagnostic Challenges in Primary Sacral Tumors and the Yield of Computed Tomography-Guided Needle Biopsy in the Modern Era. World Neurosurg..

[110] Kortman K., Ortiz O., Miller T., Brook A., Tutton S., Mathis J., Georgy B. (2013). Multicenter study to assess the efficacy and safety of sacroplasty in patients with osteoporotic sacral insufficiency fractures or pathologic sacral lesions. J. Neurointerv. Surg..

[111] Leone A., Costantini A., Guglielmi G., Settecasi C., Priolo F. (2000). Primary bone tumors and pseudotumors of the lumbosacral spine. Rays.

[112] Abraham A.P., Kingsley P.A., Negi P., Bedi H.S. (2024). Giant cell tumor of femur with single pulmonary metastasis—Yet another curative oligometastasis. J. Cancer Res. Ther..

[113] Brien E.W., Mirra J.M., Kessler S., Suen M., Ho J.K., Yang W.T. (1997). Benign giant cell tumor of bone with osteosarcomatous transformation (“dedifferentiated” primary malignant GCT): Report of two cases. Skelet. Radiol..

[114] Rose P.S. (2022). The management of sacral tumours. Bone Jt. J..

[115] Amorosa J.K., Weintraub S., Amorosa L.F., Safer J.N., Rafii M. (1985). Sacral destruction: Foraminal lines revisited. Am. J. Roentgenol..

[116] Yalcinkaya U., Doganavsargil B., Sezak M., Kececi B., Argin M., Basdemir G., Oztop F. (2014). Clinical and morphological characteristics of osteoid osteoma and osteoblastoma: A retrospective single-center analysis of 204 patients. Ann. Diagn. Pathol..

[117] Praveen B.K., Das S., Gupta M., Joshi D., Panwar H. (2023). Primary Spinal Intradural Extramedullary Ewing’s Sarcoma/Peripheral Neuroectodermal Tumour Masquerading Clinically as a Neurogenic Tumour: A Case Report and Review of Literature. Ann. Neurosci..

[118] Haibach H., Farrell C., Dittrich F.J. (1985). Neoplasms arising in Paget’s disease of bone: A study of 82 cases. Am. J. Clin. Pathol..

[119] Jurik A.G. (2023). Diagnostics of Sacroiliac Joint Differentials to Axial Spondyloarthritis Changes by Magnetic Resonance Imaging. J. Clin. Med..

[120] Renson T., de Hooge M., De Craemer A.S., Deroo L., Lukasik Z., Carron P., Herregods N., Jans L., Colman R., Van den Bosch F. (2022). Progressive Increase in Sacroiliac Joint and Spinal Lesions Detected on Magnetic Resonance Imaging in Healthy Individuals in Relation to Age. Arthritis Rheumatol..

[121] Weber U., Jurik A.G., Zejden A., Larsen E., Jørgensen S.H., Rufibach K., Schioldan C., Schmidt-Olsen S. (2018). Frequency and Anatomic Distribution of Magnetic Resonance Imaging Features in the Sacroiliac Joints of Young Athletes: Exploring ”Background Noise” Toward a Data-Driven Definition of Sacroiliitis in Early Spondyloarthritis. Arthritis Rheumatol..

[122] Ziegeler K., Eshkal H., Schorr C., Sieper J., Diekhoff T., Makowski M.R., Hamm B., Hermann K.G. (2018). Age- and Sex-dependent Frequency of Fat Metaplasia and Other Structural Changes of the Sacroiliac Joints in Patients without Axial Spondyloarthritis: A Retrospective, Cross-sectional MRI Study. J. Rheumatol..

[123] Tsoi C., Griffith J.F., Lee R.K.L., Wong P.C.H., Tam L.S. (2019). Imaging of sacroiliitis: Current status, limitations and pitfalls. Quant. Imaging Med. Surg..

